# Thermosensitivity of TREK K2P channels is controlled by a PKA switch and depends on the microtubular network

**DOI:** 10.1007/s00424-025-03089-1

**Published:** 2025-05-15

**Authors:** Sönke Cordeiro, Marianne Musinszki

**Affiliations:** Institute of Physiology, Kiel University, Kiel, Germany

**Keywords:** Thermosensitivity, Ion channel, K2P channel, TREK, Microtubule network, Temperature sensing

## Abstract

Temperature sensing is an essential component of animal perception and enables individuals to avoid painful or lethal temperatures. Many temperature sensors in central and peripheral neurons are ion channels. Here, we focus on the thermosensitive TREK/TRAAK subfamily of K2P channels—the only known K^+^ selective thermosensitive channels. The C-terminal domain is essential for the temperature activation of TREK channels, but the mechanism of temperature sensation and the nature of the temperature sensor are unknown. We studied the thermosensitivity of representatives of all K2P channel subfamilies and identified TREK-1 and TREK-2 as the only thermosensitive K2P channels, while TRAAK, the third member of the mechano-gated subfamily, showed no temperature dependence. We transferred the thermosensitivity of TREK-1 to TRAAK channels by exchanging the C-termini, demonstrating that the C-terminal domain is sufficient to confer thermosensitivity. By gradually truncating the C-terminus, we isolated a specific temperature responsive element (TRE) consisting of 18 amino acids that constitutes a unique feature in mammalian thermosensitive channels. Within this TRE lie both the binding domain for microtubule associated protein 2 (MAP2) and the PKA phosphorylation site. Pharmacological disruption of the microtubular network as well as the loss of the MAP2 binding site suppressed the temperature response, and PKA activation completely abolished temperature sensitivity. Thus, the connection to the microtubular network enables the thermosensitivity of TREK channels, which is not intrinsic to the channel itself, while the PKA-mediated phosphorylation status acts as a switch that determines if TREK channels are thermosensitive at all.

**Supplementary Information:**

The online version contains supplementary material available at 10.1007/s00424-025-03089-1.

## Introduction

Sensing temperature is of vital importance for homoiothermic animals. For the maintenance of a constant body temperature, it is sensed mainly by thermosensitive neurons in the preoptic area (POA), a part of the hypothalamus [47]. To maintain body temperature upon changes in environmental temperature and to avoid harmful temperatures, thermosensitive neurons are located in dorsal root ganglia (DRG) and trigeminal ganglia (TG) [53], where different ion channels mediate the thermosensation. A measure for temperature sensitivity of biological processes is the change of the rate of a process upon changing the temperature by 10 °C, expressed as the Q10 value. Every enzyme or ion channel has Q10 values up to 3 [32, 62]. Only very few ion channels are in fact temperature-sensitive, with Q10 values sometimes higher than 20. Major players are members of the transient receptor potential (TRP) channels, which are intensively investigated, as, e.g., disruption of the gene encoding TRPV1 leads to a strong impairment of noxious heat sensing [13, 20], while knockout of its antipode TRPM8 causes a suppression of cold sensation [7, 18, 24]. Several other TRP channels are activated by temperature changes (TRPV2, TRPV3, TRPV4, TRPM3, TRPA1, and TRPC5; [53]), but despite an intensive search, no distinct thermosensor could yet be identified in these channels.

In addition to “classical” temperature sensitive TRP channels, members of other ion channel families have also been shown to be activated by temperature changes: the voltage-dependent Na^+^ channel Na_v_1.8 [78], the Ca^2+^-activated Cl^−^ channel TMEM16 A (also known as anoctamin 1) [15], and the STIM1/Orai pair [71]. The members of the TREK/TRAAK subfamily of K2P channels are the only known thermosensitive K^+^-selective channels. Importantly, in contrast to the thermosensitive ion channels described above, activation of these K^+^ channels dampens neuronal activity [38].

While all other K^+^ channels are composed of four pore forming subunits and subsequently possess a tetrameric symmetry, K2P channels are composed of only two subunits with four transmembrane domains and two pore segments in tandem. According to their homology and similarity in activation mechanisms, the 15 members of the mammalian K2P channel family are subdivided into six subgroups—the weak inward rectifiers (TWIK), the mechano-gated (TREK/TRAAK), the alkaline-activated (TALK), the Ca^2+^-activated (TRESK), the acid-inhibited (TASK), and the halothane-inhibited (THIK) subfamily [26, 33].

All members of the TREK/TRAAK subfamily are expressed in thermosensitive neurons of the POA and the DRG and TG [41, 43, 64, 67, 72]. TREK/TRAAK channels are of essential meaning for the temperature-dependent behavior in mammals. Knockout of one or more of these channels in rodents results in significantly changed heat sensation [38, 51, 56].

TREK/TRAAK channels are regulated by many physical and cellular stimuli, i.e., temperature, mechanical stress, intracellular pH, lipid species like arachidonic acid and PIP2, as well as G protein coupled receptor (GPCR)-mediated phosphorylation and protein–protein interactions [26]. Significantly, truncated TREK-1 channels lacking the intracellular C-termini have reduced activity at rest [54] and show impaired responses to said stimuli: reduced temperature activation [41], reduced mechano-sensitivity [54], reduced sensitivity to acidification of the intracellular milieu [42], and to lipids like arachidonic acid and PIP_2_ [14]. Thus, for the action of most physiological modulators, the C-terminus is essential [6, 26]. However, the mechanism how temperature may be sensed by the C-terminal domain (CTD) to open the channel remains unclear.

Here, we investigate the mechanism of temperature-induced activation of TREK-1 K2P channels and we identify the temperature responsive element (TRE) necessary for their temperature activation. We demonstrate that thermosensitivity is not an intrinsic property of TREK-1 channels, but requires contact to the cytoskeleton that is mediated by microtubular adaptor proteins. Finally, we describe that thermosensitivity is controlled by the PKA-mediated phosphorylation status of the channel, enabling the cell to tune thermosensitivity according to its current state.

## Material and methods

### Molecular biology

Channel constructs were subcloned into the expression vector pFAW containing a CMV promotor. All K2P channels correspond to the human isoforms TREK-1 (KCNK2; NM_001017425), TREK-2 (KCNK10; NM_021161), TRAAK (KCNK4; NM_033310), TALK-2 (KCNK17; NM_031460), TRESK (KCNK18; NM_181840), TWIK-1 (KCNK1, NM_002245), TASK-3 (KCNK9, NM_001282534), and THIK-1 (KCNK13, NM_022054). For control measurements, the longer human TRAAK isoform 2 (AF247042) and the mouse TRAAK (KCNK4; NM_008431) were used. For receptor-coupled modulation of temperature sensitivity, the mouse dopamine receptor D1 (Drd1, NM_001291801) was coexpressed with the TREK-1 channel. For measurements of homomeric TWIK-1 channels, the endo-/lysosomal retrieval motif was destroyed by substituting isoleucine 293 and 294 for alanine. The MAP2 binding site was destroyed by substituting R357, K362, R363, and K364 for proline as described by Sandoz et al. [59]. Amino acid substitutions were introduced via site-directed mutagenesis with the QuikChange method (Stratagene). Channels were truncated by replacing the respective amino acid triplet by a stop codon and appropriate restriction site. Chimeric channels were constructed by the insertion of MluI restriction sites in TREK-1, TRAAK, TALK-2, and TASK-3 by silent mutation in L311/R312/V313 (TREK-1), L257/R258/V259 (TRAAK), and G271/R272/V273 (TALK-2) and by replacing Phe246 (TASK-3). Consequently, chimeras consist of TREK-1 (M1-V313)/TRAAK (V260-V393), TRAAK (M1-V259)/TREK-1 (I314-K426), TALK-2 (M1-V273)/TREK-1 (I314-K426), and TASK-3 (M1-A245)/TREK-1 (V313-K426). Heteromeric channels were constructed by concatenating TRAAK and TREK-1 with a linker sequence consisting of a XhoI restriction site followed by 10 amino acids (GGGGSGGGGS).

### Cell culture

HEK293 cells were kept in Dulbecco’s Modified Eagle’s Medium (DMEM) supplemented with 10% FCS and penicillin–streptomycin (100 U ml^−1^/100 µg ml^−1^) in a 5% CO_2_ incubator at 37 °C. The cells were transiently transfected with Lipofectamine 2000 (Invitrogen). For electrophysiological measurements, the transfected cells were trypsinized and seeded onto 10-mm coverslips at least 4 h before the experiments.

### Electrophysiology

Most electrophysiological measurements were done in the whole-cell configuration of the patch-clamp technique using an EPC10 amplifier (HEKA) and the PatchMaster software (HEKA). The cells were stimulated by a ramp protocol between − 100 and + 60 mV (1 s duration, every 5 s, holding potential − 80 mV) or by a family of rectangle pulses (1 s duration, between − 80 and + 60 mV with 20 mV increments, holding potential − 80 mV). Pipette resistances were 1–3 MΩ when filled with intracellular solution (in mM): 140 KCl, 2 MgCl_2_, 1 CaCl_2_, 2.5 EGTA, 10 HEPES, pH 7.3 with KOH. For measurements of TWIK-1 channels, K^+^ was replaced by Rb^+^ as TWIK-1 channels display nearly no K^+^ conductance. The Rb^+^-based intracellular solution was also used to measure pre-activated TRAAK channels in control measurements. Where needed, 3 mM MgATP and 0.3 mM NaGTP were included in the intracellular solution. The bath solution contained (in mM): 135 NaCl, 5 KCl, 2 MgCl_2_, 2 CaCl_2_, 10 glucose, 10 HEPES, pH 7.3 with NaOH. On-cell and inside-out measurements were performed with the following bath solution (mM): 140 KCl, 2 MgCl_2_, 2 CaCl_2_, 10 glucose, 10 HEPES, pH7.3 with KOH. All modulatory agents were added to the bath to obtain the specified final concentrations. The temperature was changed via a water-heated and -cooled bath chamber connected to a Peltier element and controlled by a temperature controller (TEM-01D, npi electronics). Fast temperature changes were obtained by heating with a halogen lamp (Osram, 15 V, 150 W) mounted in direct vicinity of the recording chamber, yielding heating rates between 0.04 and 0.5 °C s^−1^.

### Chemicals

Forskolin, phorbol-12-myristate-13-acetate, vinblastine, and H-89 were purchased from Cayman Chemical, and MgATP, NaGTP, GMP PNP, 3-isobutyl-1-methylxanthine, colchicine, cytochalasin B, arachidonic acid, and BL-1249 from Sigma-Aldrich. GTPγS was purchased from Jena Bioscience, and ML335 from Tocris Bioscience.

### Computational models

The amino acid sequences of TREK-1b (O95069-1), TREK-2b (P57789-4), TRAAK isoform 1 (Q9 NYG8-1), TALK-2 (Q96 T54-3), TASK-3 (Q9 NPC2), and THIK-1 (Q9HB14) were used to calculate homodimeric structural models with Alphafold 3 server beta [1]. The default settings were used to generate five models, and the models were analyzed and visualized with PyMOL 2.5.0 open source built [58], showing residues 46–374 for TREK-1, 46–397 for TREK-2, and 1–319 for TRAAK.

### Data analysis

All data are given as mean ± S.E.M. The fold change of temperature activation was estimated by dividing the maximally activated current by the basal current immediately before temperature elevation. Q10 values were calculated by the following equation: $\text{Q}10={(\frac{\text{I}2}{\text{I}1})}^{\frac{10}{\text{T}2-\text{T}1}}$ where T1 and T2 are temperatures at different time points and I1 and I2 are the respective currents measured at 0 mV. Graphical presentations were performed with Igor Pro (version 6.3.7.2) and Inkscape (version 1.3.2). For intra-individual data comparisons, the Wilcoxon-rank-test was performed using the Igor Pro software (WaveMetrics). Differences were considered significant if *p* < 0.05 and values of *p* < 0.05, *p* < 0.01, and *p* < 0.001 are depicted by *, **, and ***, respectively. All results are summarized in Table S1.

## Results

### Only TREK-1 and TREK-2 are thermosensitive K2P channels

**Fig. 1:**
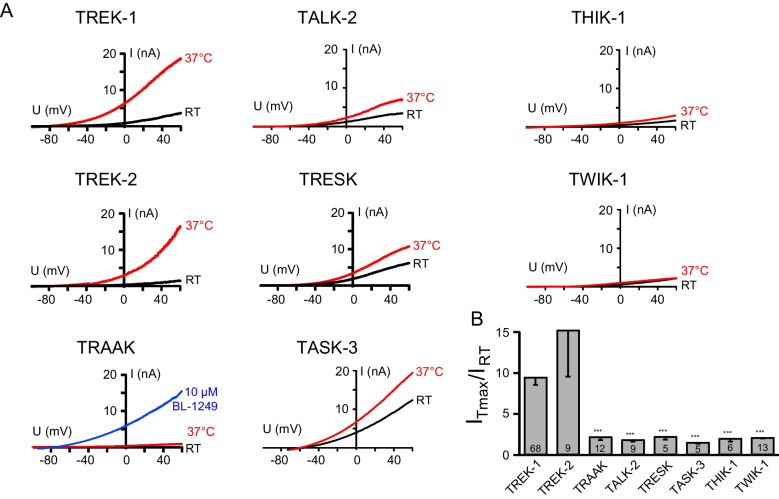
Temperature sensitivity of the TREK/TRAAK subfamily and representative members of all other K2P subfamilies. **A** Exemplary whole-cell current traces recorded from HEK293 cells transfected with the K2P channels at RT (black) and 37 °C (red). Currents were elicited with ramp protocols between − 100 and + 60 mV. As TRAAK channels showed no currents in the whole cell configuration, these channels were activated by 10 µM BL-1249 after temperature elevation. **B** Fold change of temperature activation of the tested K2P channels (as the maximal current measured at + 60 mV normalized to basal current before temperature activation)

We systematically investigated the thermosensitivity of members of all six K2P channel subfamilies, with a focus on the TREK/TRAAK subfamily for which temperature sensitivity is reported. We measured the current response of channels expressed in transiently transfected HEK293 cells upon heating, fast heating, or cooling of the bath solution. Both TREK-1 and TREK-2 channels displayed strong activation by increased temperature, while representatives of other subfamilies were not activated (Fig. 1A). The time course of temperature-dependent activation is shown in Fig. S1A for a selection of K2P channels. Q10 values for the activation of TREK-1 and TREK-2 channels reached > 20 and > 15, respectively, but varied strongly depending on the temperature and on the speed of heating (Fig. S1B). Therefore, instead of using the Q10 values, we determined the fold change of the maximal temperature-activated current across the whole temperature range.

TREK-1 and TREK-2 channels displayed a clear temperature-dependent activation with a fold change of the current of 10.53 ± 1.24 and 13.67 ± 5.25, respectively. Elevating temperature did not activate any other tested K2P channel. They showed fold changes of 1.88 ± 0.25 for TRAAK, 1.88 ± 0.21 for TALK-2, 2.25 ± 0.36 for TRESK, 1.54 ± 0.21 for TASK-3, 2.03 ± 0.41 for THIK-1, and 2.25 ± 0.19 for TWIK-1 (Fig. 1B). Surprisingly, the third member of the TREK/TRAAK subfamily, TRAAK, was not temperature sensitive. The functionality of the TRAAK channels was tested by the subsequent activation of the channels by a known pharmacological TREK/TRAAK channel activator (10 µM BL-1249). This observation conflicts with a previous study by Kang et al. [36], who reported that TRAAK channels are activated by elevated temperature. However, the study used murine TRAAK expressed in COS7 cells, which differs from the human homolog especially in the sequence of the CTD (Fig. S2A). To rule out a species-dependent effect, we also measured the temperature activation of mouse TRAAK channels. Consistent with our previous results in human TRAAK, no temperature-dependent activation was present and the fold change of 3.44 ± 0.29 was clearly below the corresponding values for TREK-1 and TREK-2 channels (Fig. S2C). To exclude a possible influence of the cellular background, we also determined the temperature sensitivity of human TRAAK channels expressed in COS7 cells, and again found no activation (fold change 2.07 ± 0.33; Fig. S2C). Further, we ruled out an influence of the longer N-terminus present in splice variant 2 of TRAAK channels, which was also not activated by temperature (Fig. S2C, fold change of 2.37 ± 0.26). To finally prevent missing a possible temperature activated current of TRAAK channels because of their very low open probability in the basal state, we measured with Rb^+^ in the pipette solution, which opens the selectivity filter [61]. Also under these conditions, TRAAK channels showed no thermosensitivity with a fold change of 1.51 ± 0.11 (Fig. S2B).

It is worth noting that within the representatives of other K2P subfamilies, only TWIK-1 mediated currents also responded to elevated temperature. However, instead of being activated, TWIK-1 channels became inactivating upon depolarization with continuous heating to 37 °C (Fig. S3), leading to a current decrease below the initial current at room temperature. This phenomenon has also been described for TWIK-2 channels [55].

Thus, we find that TREK-1 and TREK-2 channels are the only K2P channels that are activated by elevated temperatures.

### Temperature sensitivity is not intrinsic to TREK-1 channels

**Fig. 2:**
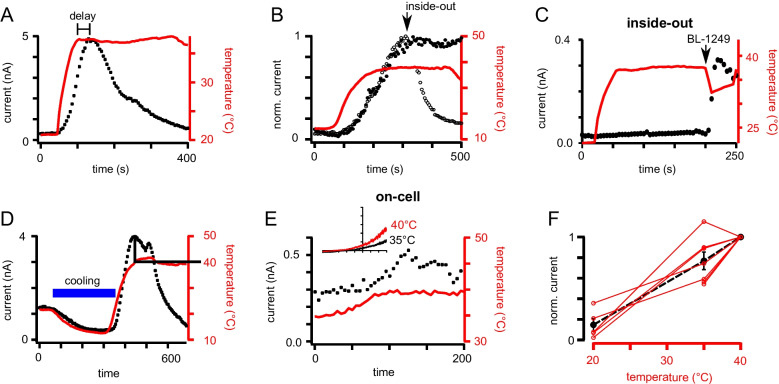
Temperature response of TREK-1 channels depends on the recording mode during activation. **A** Time course of temperature activation in whole-cell recordings of TREK-1 channels. Upon fast temperature elevation, a delay between the maxima of temperature and current is visible. Currents were elicited with a ramp protocol (− 100 to + 60 mV) and currents measured at + 60 mV were plotted over time. **B** Comparison of the time course of temperature activation in the on-cell mode (filled circles) with the time course in inside-out configuration, showing the inactivation upon patch excision after reaching maximal activation (open circles; arrow marks moment of excision). Currents were measured with the ramp protocol in symmetrical K^+^ solutions. **C** Time course of currents measured in the inside-out mode with temperature elevation. As no currents were activated, the channels were activated by 10 µM BL-1249 after temperature elevation as a control (arrow). **D** Time course of temperature activation measured as in **A** but including a cooling step to ~ 10 °C before temperature elevation. **E** On-cell recording of temperature activation in the physiological relevant temperature range (35 to 40 °C). Currents were measured as in **B**. The inset shows exemplary ramps measured at 35 °C (black) and 40 °C (red) from the same patch. **F** Current response of measurements like in **E** plotted against the temperature. The red circles and lines show individual cells, the black circles and line the mean and S.E.M. of the five cells

The temperature-induced current increase over time disclosed two features: TREK channels activated slower than the temperature rise, and currents decreased upon further holding of the temperature in the whole-cell configuration (Fig. 2A). To show that the channels were still functional even after a complete current decrease, we supplied BL-1249 (Fig. S4C). The delays in time courses of current activation were nearly independent from the heating speed, reaching their maxima at the earliest 1 min after starting the temperature rise (Figs. S1A, S4 A; compare time courses with a heating rate of 0.1 °C s^−1^ and 0.25 °C s^−1^). Consequently, also the temperatures at maximal current depended significantly on the heating speed (Fig. S4B; note maximal currents at 36 °C and 49 °C). We tested if the activation state of the selectivity filter could influence this temperature response (Fig. S5). Preactivation of the C-type gate by the T157 C mutation or activation by ML335, which render the channel mostly insensitive to voltage and many other stimuli [40, 61], did not alter the time course of the temperature response; as expected, no additional current increase was measured in the fully open G152I mutant [6, 40]. TREK-1 channels are also sensitive to cooling below room temperature, which results in current decrease (Fig. 2D). Cooling of the cells led to a nearly complete closure of TREK-1 channels, and subsequent heating activated the channels as usual with a maximum at 40 °C (Fig. S4B). With this temperature protocol (i.e., cooling to 10 °C before heating), the maximal current was also reached after about 2 min, showing that no specific threshold for temperature activation exists within the applied temperature range.

To test the possible involvement of intracellular factors in the temperature activation mechanism, we measured TREK-1 channels in different patch configurations. Importantly, the observed thermosensitivity was preserved upon prolonged heating in on-cell measurements, where the current maximum was stable at constant temperature (Fig. 2B; fold change 8.50 ± 1.17). In contrast, after excision of the patches, yielding the inside-out configuration, channels immediately and completely lost their temperature sensitivity (Fig. 2B). When measurements were done in the inside-out configuration from the beginning, the channels display no thermosensitivity, as reported before [36, 41] (Fig. 2C; fold change 1.2 ± 0.25), but still were functional as they were activated by other stimuli, i.e., arachidonic acid or BL-1249 (Fig. 2C). Hence, together with the delay in the onset of current activation after temperature rise, this dependence on cell integrity suggested that temperature activation is not an intrinsic channel property.

Since we so far used a broad temperature range between 10 and 50 °C to characterize temperature activation of the current, the question arose whether TREK channels are activated in the physiologically relevant temperature range. Therefore, we measured TREK-1 channels in the on-cell configuration at 35 °C and further raised the temperature to about 40 °C. Indeed, a small but clear temperature-dependent current increase was induced (Fig. 2E). Plotting the current increase induced by a temperature rise from room temperature to 35 °C and the rise from 35 to 40 °C yielded a linear relationship, meaning that the temperature sensor of TREK-1 channels was equally active between 21 and 35 °C and at temperatures around normal body temperature, i.e., 35–40 °C (Fig. 2F).

### The C-terminal domain of TREK-1 channels contains a temperature-responsive element

We intended to identify the channel region responsible for the thermosensitivity in TREK channels. It has been reported previously that the C-terminus of TREK-1 channels is indispensable for their temperature activation, and a decoupling between the CTD and the pore-forming core also leads to the loss of thermosensitivity [6, 41].

We have shown above that TRAAK channels were not thermosensitive (Figs. 1 and S2). A sequence comparison of both C-termini showed that only the proximal part up to the known PKA site in TREK-1 channels [27] is similar within the subfamily, and that the TRAAK sequence proceeds with a proline-rich region (Fig. S6A) that is not expected to form an α-helix. We additionally compared Alphafold 3 models of TREK-1 and TRAAK channels, which show that TREK-1 channels with high probability have an elongated helical C-terminus up to N372, while the TRAAK C-terminus is unordered distal to A290, corresponding to the beginning of the PKA recognition site in TREK-1 (Fig. S6B).

**Fig. 3:**
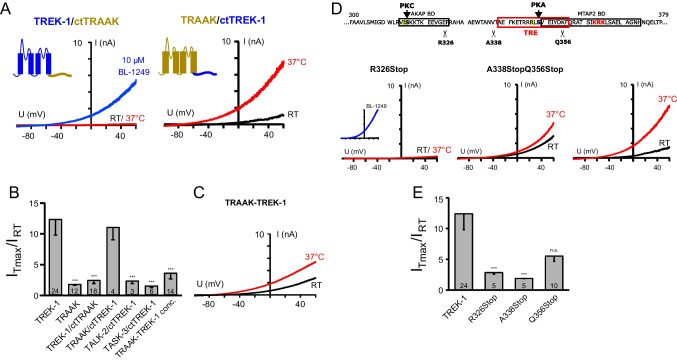
Identification of the temperature responsive element (TRE) in the C-terminus of TREK-1 channels. **A** Exemplary current traces of HEK293 cells transfected with chimeric constructs of TREK-1 core channels with C-termini of TRAAK channels (TREK-1/ctTRAAK, left) and vice versa (TRAAK/ctTREK-1, right) measured at RT (black trace) and 37 °C (red trace). 10 µM BL-1249 (blue trace) was used to show the functionality of the chimeric TREK-1/ctTRAAK channels. **B** The bar diagram shows the fold change of channel activation upon temperature elevation for TREK-1 and TRAAK wild type and different chimeric channels. **C** Exemplary current traces of concatenated TRAAK-TREK-1 channels measured at RT (black trace) and 37 °C (red trace). **D** Part of the TREK-1 C-terminal sequence with the known PKC and PKA phosphorylation sites. The binding motifs for AKAP150 and MAP2 are marked with black boxes, the identified TRE with a red box. The scissors indicate the sites of truncation. Below, exemplary current traces of the truncated TREK-1 channels are shown (black traces: at RT; red traces: at 37 °C; inset: control with 10 µM BL-1249). **E** Bar diagram showing the fold change of activation upon temperature elevation for truncated TREK-1 channels

In order to prove the importance of the TREK C-terminus for temperature activation, we constructed chimeric channels where the entire C-termini were swapped between the two channels (after residues V313 of TREK-1 and V259 of TRAAK), yielding the constructs TREK-1/ctTRAAK and TRAAK/ctTREK-1, respectively (Fig. 3A). Indeed, the swapping of the C-termini led to an exchange of their thermosensitivity. While TREK-1/ctTRAAK channels entirely lost their thermosensitivity with the introduction of the TRAAK C-terminus (fold change 2.53 ± 0.60), thermosensitivity was transferred to TRAAK/ctTREK-1 channels with the C-terminus of TREK-1, with an activation comparable to the TREK-1 wild-type channel (Fig. 3A, B; fold change 12.45 ± 2.65 for WT TREK-1 and 10.77 ± 2.01 for TRAAK/ctTREK-1). This transfer of the thermosensitivity by connecting the respective pore forming core to the TREK-1 C-terminus was only effective within the TREK/TRAAK subfamily, as other chimeric channels failed to yield temperature sensitive channels (i.e., TALK-2 or TASK-3 core channels with TREK-1 C-terminus; fold change of 2.81 ± 0.75 for TALK-2/ctTREK-1 channels and 2.13 ± 0.46 for TASK-3/ctTREK-1 channels; Fig. 3B).

As K2P channels form dimers to build functional channels, homomeric channels have two identical C-termini. However, it is known that TREK/TRAAK channels can form heteromeric channels within their subfamily [9, 39]. We intended to investigate the influence of one native thermoinsensitive C-terminus on the thermosensitivity of the whole channel and constructed concatenated channels of TREK-1 subunits with temperature-insensitive TRAAK channel subunits. Interestingly, concatemers of TRAAK and TREK-1 subunits were not activated by temperature, providing an indication that one TREK-1 subunit is not enough to confer temperature sensitivity to heterodimeric channels (fold change 3.40 ± 0.81, Fig. 3B, C).

To further narrow down the region responsible for thermosensitivity, we gradually truncated the C-terminus of TREK-1 (Fig. 3D). All truncated constructs were still functional and regulated by BL-1249 (inset for R326Stop in Fig. 3D). Truncating the complete TREK-1 C-terminus at arginine 326 (R326Stop) led to a complete loss of thermosensitivity (fold change of 2.9 ± 0.33, Fig. 3D, E). The thermosensitivity was still lost upon truncation at alanine 338 (A338Stop, fold change 1.93 ± 0.07). In contrast, the truncation of only the last 70 amino acids (Q356Stop) had little effect on the thermosensitivity of TREK-1 channels (fold change 5.64 ± 0.91). Thus, the stretch of 18 amino acids from A338 to F355 must comprise the temperature responsive element of TREK-1 channels (TRE hereinafter).

### The PKA-mediated phosphorylation state regulates thermosensitivity

In addition to the known phosphorylation sites for PKC and PKA, the TREK-1 C-terminus contains binding sites for the interacting proteins AKAP5 (AKAP150 in mouse) and microtubule associated protein 2 (MAP2) close-by (Fig. 3D; [59, 60]). A sequence analysis of the TRE revealed that two of these previously described modifiable elements lie within this region: the PKA phosphorylation site at S348 and the MAP2 binding domain from E350-Q375. TREK-1 channels are modulated by phosphorylation by PKC and PKA at two serines (S315 and S348; [48, 54]). Therefore, we sought to investigate the influence of the kinases on the thermosensitivity of TREK-1 channels using pharmacological modulators. The PKA site S348 (S333 in the shorter isoform) in TREK-1 is also present in the other thermosensitive K2P channel TREK-2 (here S364), while it is absent in the thermoinsensitive TRAAK channels (Fig. S2A). In contrast, the PKC phosphorylation site S315 (S300 in the shorter isoform) is present in TREK-2 (S331) as well as TRAAK (S287), though it has been described to be not functional in TRAAK channels [28].

**Fig. 4:**
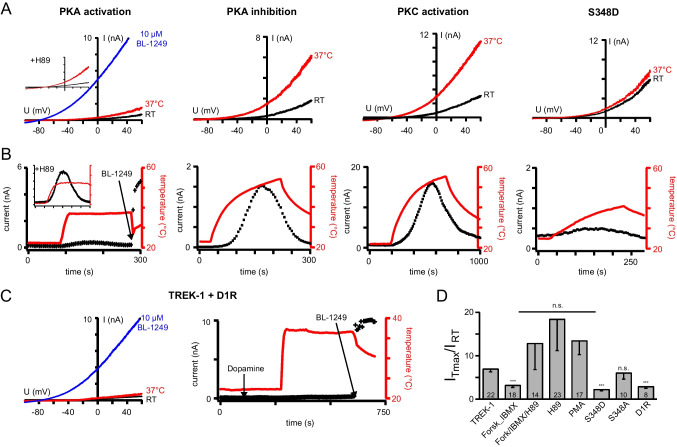
PKA phosphorylation specifically suppresses thermosensitivity in TREK-1 channels. **A** Exemplary basal and temperature activated TREK-1 currents after pharmacological kinase modulation. The inset shows an example of a cell preincubated with H-89 before the application of Forsk/IBMX (black traces: at RT; red traces: at 37 °C). **B** Time courses of temperature activated currents with kinase modulation as shown in **A**. **C** Exemplary current traces and time course of TREK-1 currents co-expressed with the G protein coupled dopamine receptor 1 (Drd1) exposed to dopamine and temperature elevation. As no currents were activated, the channels were activated by 10 µM BL-1249 after temperature elevation (blue trace/arrow). **D** Bar diagram showing the fold change of temperature activation after kinase modulation or Drd1 receptor activation

Activation of PKC by phorbol-12-myristate-13-acetate (PMA; 1 µM), though reducing the basal current, had little effect on temperature activation (fold change 13.50 ± 3.25; Fig. 4). In contrast, the activation of PKA by 10 µM forskolin/100 µM 3-isobutyl-1-methylxanthine (Forsk/IBMX) dramatically inhibited the basal current and additionally abolished the temperature activation of TREK-1 channels (fold change 3.21 ± 0.54; Fig. 4). This was not due to dysfunction of the channels as they were still activated pharmacologically by BL-1249. Preincubation with the PKA inhibitor H-89 completely prevented this inhibitory effect of Forsk/IBMX on the thermosensitivity (fold change 12.89 ± 6.07; Fig. 4 insets). Inhibition of PKA by 1 µM H-89 alone had no effect or even augmented the temperature activation (fold change of 18.00 ± 6.99; Fig. 4D). Hence, thermosensitivity of TREK-1 channels is specifically controlled by the PKA phosphorylation status, and PKA activation is sufficient to disable a temperature response. Importantly, this PKA-mediated phosphorylation itself could not constitute the temperature-dependent step, as the thermosensitivity was preserved after inhibition of PKA by H89.

In addition, we mutated the PKA phosphorylation site in TREK-1 channels either to aspartate to mimic the phosphorylated state (S348D) or to alanine to mimic the dephosphorylated state (S348A). Like the activation of PKA by Forsk/IBMX, the S348D mutation led to a dramatic decrease in basal current and completely abolished the temperature activation, supporting the importance of the degree of PKA-mediated phosphorylation for the thermosensitivity of TREK channels (fold change: 2.27 ± 0.34; Fig. 4). Likewise, in agreement with the inhibition of PKA by H-89, the S348A mutation had no effect on the thermosensitivity (fold change 6.07 ± 1.46; Fig. 4D).

Finally, we activated PKA via a physiological pathway by coexpression of dopamine receptor 1 (Drd1) as an example of a G_αs_ coupled receptor. Consistently, PKA activation via the receptor mediated cAMP synthesis prohibited temperature activation and reduced temperature activated currents compared to cells not expressing Drd1 (fold change 2.92 ± 0.43, Fig. 4C, D).

### Contact to the cytoskeleton is necessary for temperature activation

**Fig. 5:**
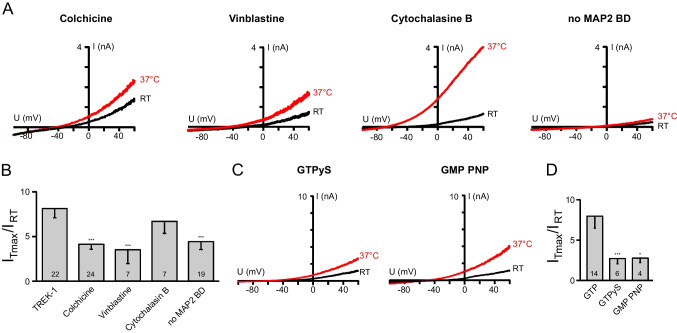
Thermosensitivity of TREK-1 channels depends on the connection of the C-terminus to the microtubular network. **A** Exemplary temperature stimulated TREK-1 currents after pharmacological modulation of the cytoskeleton or mutation of the MAP2 binding motif (black traces: at RT; red traces: at 37 °C). **B** The bar diagram shows the fold change of temperature activation from measurements as in **A** analyzed at + 60 mV. **C** Current traces of TREK-1 channels measured with the non-hydrolysable GTP analogs GTPγS or GMP PNP in the pipette solution (black traces: at RT; red traces: at 37 °C). **D** Bar diagram showing the fold change of temperature activated currents in the presence of GTP and its non-hydrolysable analogs

An interaction between MAP2 and the MAP2 binding site in TREK-1 (E350-Q375) has been shown by co-immunoprecipitation experiments with synaptosomal brain proteins [59]. As MAP2 links the associated interaction partners to the cytoskeleton, the overlap of the MAP2 binding site with the TRE (E339-Q356) suggested a possible involvement of MAP2 mediated contact between TREK-1 channels and the cytoskeleton in temperature sensing. To identify which of the different parts of the cytoskeleton is linked to the TREK-1 C-terminus, we used colchicine (0.5 mM) and vinblastine (50 µM) to disrupt the microtubule network and cytochalasin B (25 µM) to disrupt the polymerization of the actin cytoskeleton (Fig. 5A, B).

Interestingly, interference with the actin cytoskeleton had only a minor effect on temperature activation (fold change 6.74 ± 1.39), while the disassembly of the microtubular network led to the suppression of the temperature activation (fold changes 4.18 ± 0.61 and 3.56 ± 1.58 for colchicine and vinblastine, respectively).

As for the formation of microtubule the hydrolysis of GTP bound to tubulin is necessary, we also included non-hydrolysable GTP analogs in our pipette solution. Neither in the presence of GTPγS nor with GMP PNP instead of GTP, we measured any temperature activated TREK-1 current (Fig. 5C, D; fold change for GTP 8.02 ± 1.53, for GTPγS 2.76 ± 0.7, and for GMP PNP 2.74 ± 0.68).

Sandoz et al. [59] have shown that an exchange of the amino acids corresponding to R357, K362, R363, and K364 to proline in the C-terminus of TREK-1 channels impedes the binding of MAP2. We used this mutant with destroyed MAP2 binding site to test for the contribution of the TREK-1/MAP2 interaction to the temperature sensitivity. This significantly reduced the thermosensitivity of the channels (Fig. 5A, B; fold change 4.47 ± 0.92), again suggesting that the integrity of the microtubules is necessary for temperature sensitivity of TREK-1 channels.

## Discussion

In this study, we report the first localized TRE in a mammalian thermosensitive ion channel. We identified a region in the C-terminus of the channel as being indispensable for its thermosensitivity. Especially, the PKA phosphorylation site and the MAP2 binding site are essential for temperature-activation of TREK channels.

In mammals, temperature perception is mainly mediated by temperature-sensitive ion channels, primarily by members of the TRP channel family. Despite intensive efforts, the molecular basis for their thermosensitivity has not yet been fully elucidated. Two general mechanisms of thermosensitivity are discussed: global vs. localized mechanisms [3]. A hypothesis that represents a global mechanism is the temperature-dependent change in specific heat capacity of a channel [17, 75]. This would enable temperature-dependent gating transitions without the need of specific TREs, and the overall heat capacity of open and closed states could also be altered by, e.g., binding events, mutations, or deletions in distant parts of the channel. For example, Chowdhury et al. [16] were able to obtain a temperature-gated Shaker K_v_ channel by rational design of amino acid positions involved in gating transitions, emphasizing the importance of solvation changes for the heat capacity of the channel. In contrast, a localized mechanism would require the existence of a defined TRE that controls the channel gate in response to its stimulus. Examples of both can be found in temperature-sensitive ion channels. In most thermoTRP channels, changes in very different parts of the protein impede thermosensitivity, and no distinct temperature-sensing element could be identified to date. In the well-studied TRPV1 channel, the pore region seems to be essential as mutations or deletions in the extracellular part of S6 and the pore [30, 31], or the pore turret [19, 73] impair its temperature sensitivity. Furthermore, Zhang et al. [77] were able to transfer the temperature sensitivity from TRPV1 to the Shaker K_v_ channel simply by exchanging their pore domains, supporting the idea of a localized thermosensor in these channels. Contradictory to this, intracellular parts of the TRP channels, e.g., the membrane proximal domain between the ankyrin repeat domain (ARD) and TM1 [74] and the C-terminus [10], have also been found to influence the temperature sensitivity in TRP channels. In the bidirectional heat- and cold-activated TRPA1 channel, deletions in the N-terminal voltage sensing like domain (VSLD) abolished exclusively heat sensitivity, but cool sensitivity seemed to be distributed to linker and CTD with possible contributions of N-terminal ARD and the VSLD domains [35, 46]. In addition, recent cryo-EM structures of TRPV1 and TRPV3 channels generated at different temperatures also point to a global temperature activation in these channels [37, 49, 63]. To our knowledge, a clearly delimited area of the protein which represents a true localized mechanism of temperature sensitivity could only be identified in one ion channel: In the bacterial Na_v_ channel of *Silicibacter pomeroyi*, a short ≈14 amino acid stretch in a metastable domain connecting the CTD to the pore was shown to control temperature-dependent voltage gating depending on its degree of disorder [4].

Several studies have shown that TREK channels are temperature sensitive, but the mechanism of temperature activation remained unknown [6, 36, 41, 51, 56, 67]. In agreement with previous investigations, we measured Q10 values up to 20 for TREK-1 and TREK-2 channels [36, 41]. All other tested representatives from the different K2P subfamilies were not temperature activated and exhibited Q10 values below 4. Surprisingly, the third member of this K2P subfamily, TRAAK, which is closely related to TREK-1 and TREK-2, was not activated by temperature. It has been postulated that TRAAK channels are thermosensitive, albeit with a higher activation threshold (31 °C) than TREK-1 and TREK-2 channels [36]. However, we did not find any thermosensitivity upon heating to temperatures of 40 °C and more though we tested human and murine TRAAK channels in both expression systems (HEK293 and COS7 cells).

The C-terminus of TREK-1 channels is of great importance for the integration of various stimuli in addition to thermosensitivity [41], including intracellular pH [42], polyunsaturated fatty acids [54], PIP_2_ [14], and phosphorylation [27, 48], and it provides binding sites for interacting proteins [59, 60]. By swapping the TRE-containing C-terminus of TREK-1 with that of the non-thermosensitive TRAAK channel, we were able to transfer the thermosensitivity to the TRAAK channel, and vice versa make TREK-1 channels temperature-insensitive. This shows that the TREK-1 C-terminus is not only necessary but sufficient for temperature sensitivity within the TREK/TRAAK subfamily. The comparison of the C-termini of the temperature-sensitive TREK-1 and TREK-2 channels with that of TRAAK channels revealed that only TREK-1 and TREK-2 channels have particularly elongated M4 helices (Fig. S6B, C). The disruption of the α-helical structure in the C-terminus of TRAAK channels is right in the center of the TRE, suggesting that these may be a prerequisite for its function. Interestingly, the heteromeric channel formed of one TRAAK subunit and one TREK-1 subunit was not temperature-sensitive, suggesting that two TREK C-termini are needed for temperature activation. In good agreement, Bagriantsev et al. [6] have shown that a concatemer of two TREK-1 subunits where one had an uncoupled C-terminus had a blunted temperature response. However, for other activating stimuli via the C-terminus, only one C-terminus is sufficient (e.g., PKA, pH_i_, PLD2 [9, 39]) suggesting that distinct mechanisms exist for stimuli activating the channel via the CTD. This has great impact on the properties that may emerge in native heteromeric channels in the respective tissue.

By further step-wise truncation, we consequently isolated the TRE critical for temperature activation in the TREK-1 CTD to 18 amino acids (A338-F355). Importantly, this helical region includes the PKA phosphorylation site and overlaps with the MAP2 binding site which connects the TREK-1 C-terminus to the cytoskeleton.

In general, temperature-sensitive channels differ in whether they are intrinsically temperature-sensitive or require a co-factor. For example, TRPV1, TRPV3, or TRPM8 channels are still regularly temperature-sensitive after reconstitution in lipid bilayers, so it can be assumed that these channels are intrinsically temperature-dependent [12, 49, 76]. In contrast, TRPV4 channels, like TREK-1 channels, lose their temperature sensitivity when their cellular context is removed [69]. The fact that temperature activation is absent when TREK channels are measured in excised patches [36, 41], and our observation that temperature activated currents decrease in whole-cell measurements and upon inside-out patch excision is consistent with the loss of intracellular factors that convey thermosensitivity. Furthermore, the time delay of activation after the onset of temperature rise seen in TREK-1 may hint at the involvement of additional processes, while TRPV1 activity closely follows the rate of temperature elevation [12]. To shed light on the mechanism of TREK-1 temperature activation, we investigated the role of different components of the cytoskeleton as putative MAP2 binding partners as well as the relevance of kinase phosphorylation. Appropriately, we found that microtubule dynamics determined the temperature activation of TREK-1 channels. When we pharmacologically manipulated different cytoskeleton constituents, only the disruption of the microtubules or interference with their synthesis, i.e., by non-hydrolysable GTP or GTP-PNP, impeded thermosensitivity.

Temperature has a strong influence on the equilibrium between catastrophe and rescue of microtubule (how disassembly and assembly are called), with higher temperatures promoting the rescue. Significantly, MAP proteins regulate dynamics of microtubules in a process called selective stabilization and protect, e.g., from cold-induced disassembly (reviewed by [5, 21, 22]). The family member MAP2 is expressed in neurons and was shown to co-immunoprecipitate with TREK-1 channels in a lysate obtained from mouse brains [2, 59]. Our results show that the disconnection of MAP2 and TREK-1 channels by mutating the MAP2 binding region in the C-terminus of TREK-1 channels completely suppressed the thermosensitivity. This means that a change in temperature leads to a modified connection between the TRE of TREK-1 and the microtubular network, which then causes the activation of TREK-1 channels with increasing temperatures, and their closure upon cooling.

Interestingly, beyond modulation of the fundamental temperature dependence of assembly/disassembly, the family member MAP6 has been described as temperature sensor that binds to microtubules at lower temperatures [23]. Mechanistically, a linear unfolding of a specific domain from beta structures at high temperatures to a disordered state at low temperatures was shown, a structural feature which is also present in the MAP2/tau subfamily [23].

Furthermore, this non-intrinsic temperature activation of TREK-1 channels is modulated by the current state of the cell, reflected by PKA activity. The pharmacological or receptor-mediated activation of PKA specifically abolished temperature activation, while PKC activation, though also inhibiting the basal current, had no effect on the temperature dependence of the channel. The prevention of temperature activation by PKA-mediated phosphorylation has been postulated previously by Maingret et al. [41], who used cAMP to inhibit the channels at different temperatures. The inhibition was absent in the PKA site mutant S333A (S348A in the TREK-1 isoform used in this study) and was interpreted as a reversal of the temperature activation by the activation of PKA. However, the PKA-mediated phosphorylation itself cannot be the temperature sensitive step, as otherwise the inhibition of PKA by H-89 or the dephosphorylation mimicking mutant S348A should also have led to the suppression of thermosensitivity in our experiments. Thus, the phosphorylation of TREK-1 channels by PKA only changes the status of the channel, rendering it insensitive to temperature activation. Phosphorylation operates as a molecular switch, which allows the cell to adapt the TREK-1 temperature sensitivity by changing the degree of PKA-mediated phosphorylation, thereby coupling it to active signaling pathways.

The contact between the C-terminus of TREK-1 channels, the cytoskeleton, and the catalytic domain of PKA is likely mediated by MAP2 [45, 52]. Therefore, MAP2 is a key player in the temperature-dependent activation of TREK-1 channels and critical in the recruitment of PKA to the TREK-1 C-terminus. When S348 is dephosphorylated, the channel is connected to the microtubular network and temperature-sensitive; upon phosphorylation, the connection may be altered or lost, and the channel is no longer sensitive to temperature.

It has been shown that in TREK channels, mechanoactivation induces a conformational change from a low activity “down” conformation to a high activity “up” conformation, where the proximal CTD is close to the inner membrane leaflet [11, 25]. This has been postulated also for other stimuli including temperature and dephosphorylation [33, 44, 66]. One may speculate that the MAP2-mediated contact to the cytoskeleton stabilizes TREK-1 channels in the temperature-induced high activity “up” conformation [44]. Phosphorylation, however, would favor the low-activity “down” state [66], switching off temperature activation.

TREK channels are expressed in both DRG/TG neurons and thermosensitive neurons of the POA [41, 43, 64, 67, 72] and may therefore contribute to peripheral and central thermosensation. In DRG/TG neurons, TREK-1 is the counterpart of TRPV1 channels, and accordingly, these neurons would be medium temperature sensitive at rest, as their stimulation by TRPV1 activation would be dampened by TREK-1 channel activation. However, TRPV1 channels are sensitized to temperature changes by PKA-mediated phosphorylation, e.g., in ongoing inflammation [8, 29], where mediators like prostaglandins activate PKA [34], and, correspondingly, many studies have connected heat hyperalgesia during inflammation to TRPV1 channels [68]. Conversely, TREK-1 channels are desensitized to temperature changes by PKA-mediated phosphorylation ([41] and present study), and the suppression of TREK channel thermosensitivity may as well contribute to prostaglandin mediated hyperalgesia. Thus, the firing threshold of thermoreceptor fibers might be controlled by a balance of the phosphorylation states of both antagonistic channels.

In the POA, the core body temperature is closely monitored, but the set point for body temperature is not fixed; e.g., during sleep, the set point is lowered, whereas it is elevated in fever [50, 65]. Fever induced by pathogens like bacteria is mediated by pyrogens as prostaglandin E2, which suppresses the cAMP/PKA pathway through G_αi_ coupled EP_3_ prostaglandin receptors. It has been established that EP_3_ receptor activation results in the decrease of the firing rate in the subset of “warm-sensitive” POA neurons, leading to subsequent thermogenesis [47, 57, 58]. Model simulations of such neurons showed that the expression of temperature-activated TREK channels is capable of reducing their firing rate in the physiological temperature range (32–40 °C; [70]). Therefore, TREK thermoactivation upon suppression of PKA-mediated phosphorylation could contribute to the change in temperature setpoint of POA neurons under inflammatory conditions.

## Supplementary Information

Below is the link to the electronic supplementary material.

Supplementary file1 (PDF 1881 KB)

## Data Availability

No datasets were generated or analysed during the current study.
