# Supplementary material for: Thermosensitivity of TREK K2P channels is controlled by a PKA switch and depends on the microtubular network
Source: Pflugers Arch. 2025 May 15;477(7):953–66. doi: 10.1007/s00424-025-03089-1 (PMC12152049; doi:10.1007/s00424-025-03089-1)
Supplement: Supplementary file 1 — Supplementary file1 (PDF 1881 KB) [file 424_2025_3089_MOESM1_ESM.pdf]

Supplementary material to:

Thermosensitivity of TREK K2P channels is controlled by a PKA switch and depends on the microtubular network

Sönke Cordeiro[1]*, Marianne Musinszki[1]

ORCID ID SC: 0000-0002-1049-8303; MM: 0000-0002-5597-6735

*To whom correspondence should be addressed: s.cordeiro@physiologie.uni-kiel.de

1: Institute of Physiology, Kiel University, Kiel, Germany

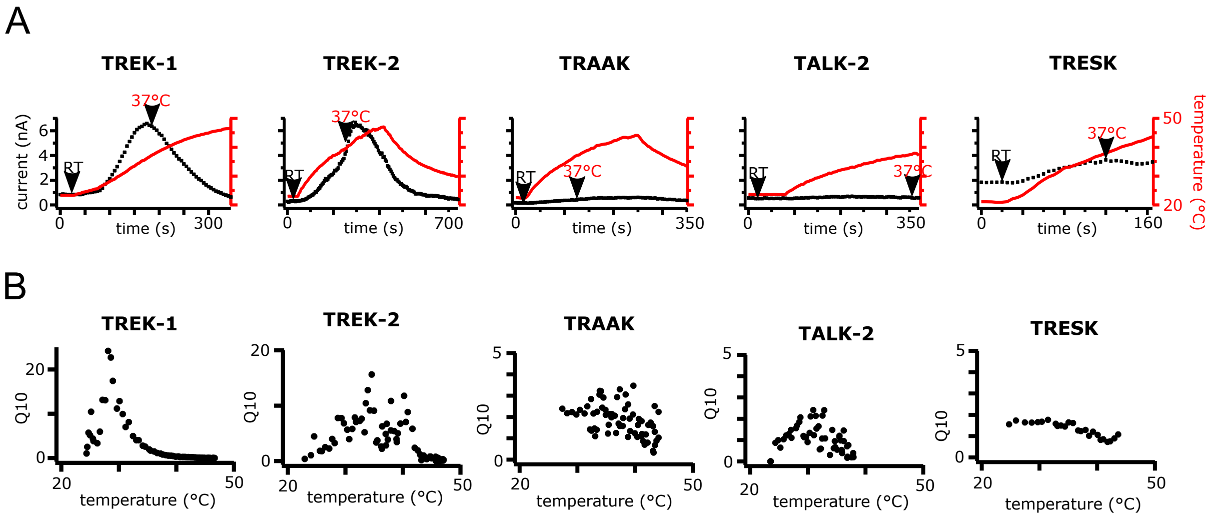

Figure S1 The Q10 values of TREK channels are temperature-dependent. **A** Time courses of the current response of different tested K2P channels to temperature elevation. The currents were elicited with a ramp protocol and plotted at 0 mV. The red line shows the time course of the monitored bath temperature; the arrow indicates the current at 37 °C. **B** Exemplary Q10 values for the currents shown in A. The values were calculated for each pair of ramps during the temperature elevation, yielding Q10 values up to 20 for TREK-1 and TREK-2. For the other tested K2P channels maximal Q10 values of 3 were calculated (note the different scaling on the y axes).

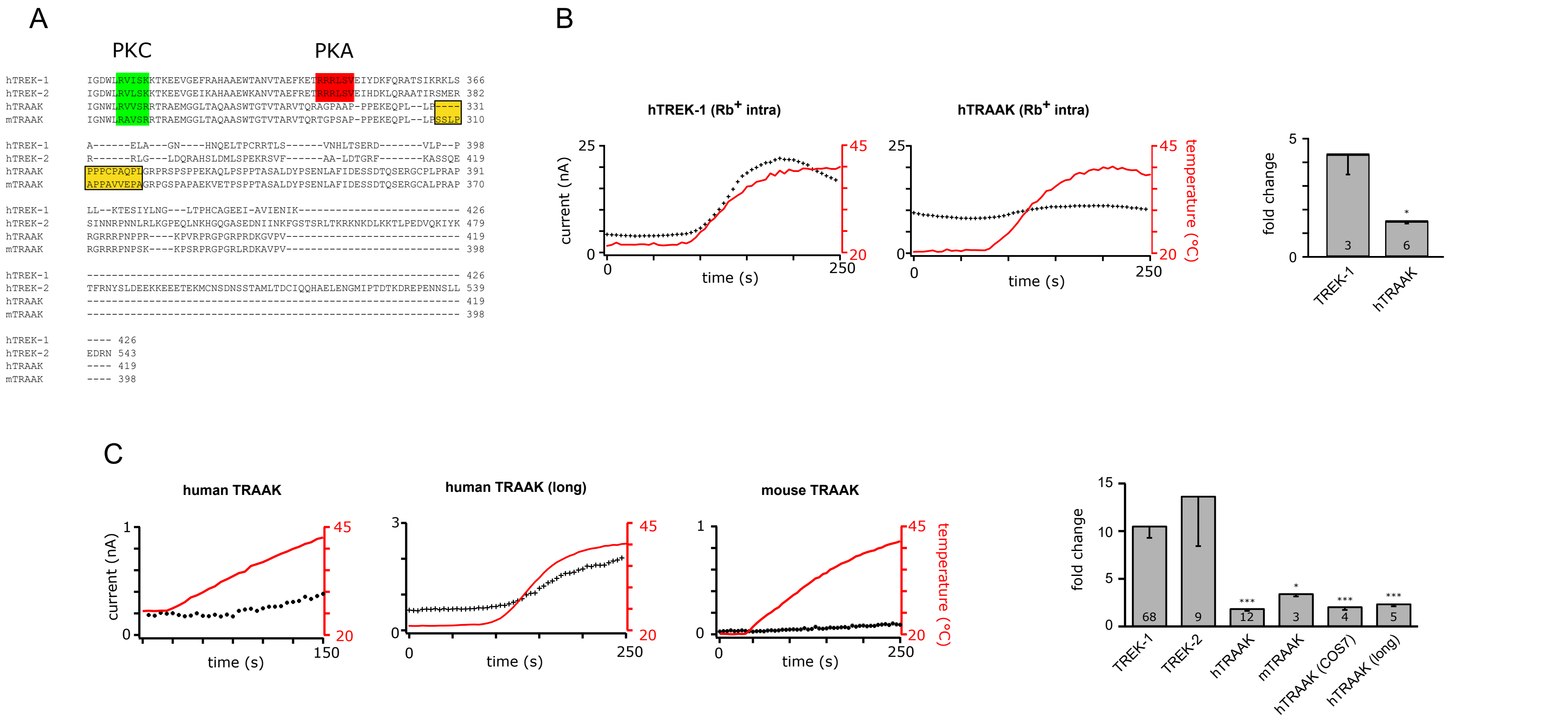

Figure S2 Neither human nor murine TRAAK channels are thermosensitive. A Sequence alignment of the C termini of human K2P members of the TREK/TRAAK subfamily and mouse TRAAK. Highlighted are the phosphorylation sites for PKC (green, conserved in TRAAK) and PKA (red, not conserved in TRAAK). In addition, a stretch of 9 amino acids in human TRAAK (13 amino acids in mouse) is highlighted that is not conserved between human and murine TRAAK (orange). **B** Time course of current changes upon temperature elevation in human TRAAK and TREK-1 channels with Rb^+ in the intracellular solution. Currents were measured with a ramp protocol and the currents at +20 mV were plotted. Red lines: changes in bath temperature measured in °C. Right: Fold change of human TREK-1 and TRAAK currents induced by temperature elevation cmeasured with Rb^+ in the intracellular solution. **C** Time course of current changes upon temperature elevation in human and mouse TRAAK channels. Currents were measured with a ramp protocol and the currents at 0 mV were plotted. Red lines: changes in bath temperature measured in °C. Right: Fold change of TREK-1 and TREK-2 currents induced by temperature elevation compared to human and mouse TRAAK channels expressed in HEK293 and COS7 cells.

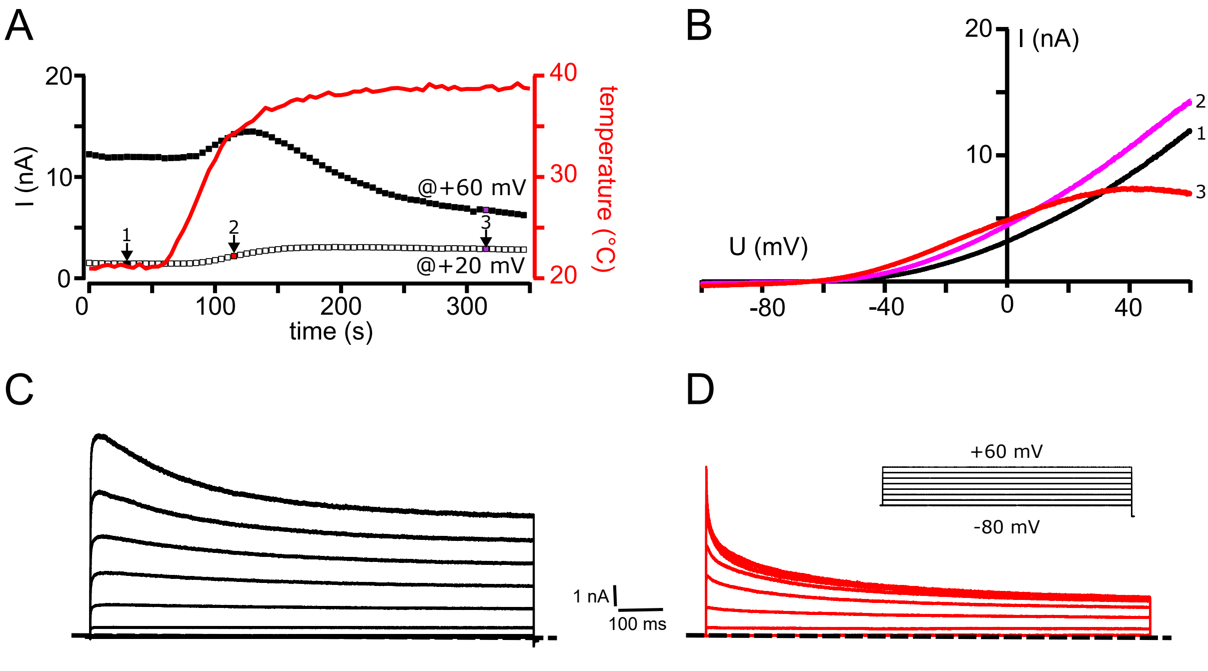

Figure S3 Temperature sensitivity of TWIK-1 channels. A Current response of TWIK-1 channels expressed in HEK293 cells to temperature elevation, measured with a ramp protocol (-100 to +60 mV; with Rb^+ instead of K^+ in the pipette solution). Red line: monitored temperature in the bath (filled squares: current at +60 mV; open squares: Current at +20 mV). **B** Current traces recorded with the ramp protocol at the time points indicated in A (1: at RT; 2: directly after temperature elevation; 3: with sustained temperature stimulation). **C** and **D** Current responses to families of rectangle pulses from -80 to +60 mV measured at RT (**C**) and at 37 °C (**D**), respectively.

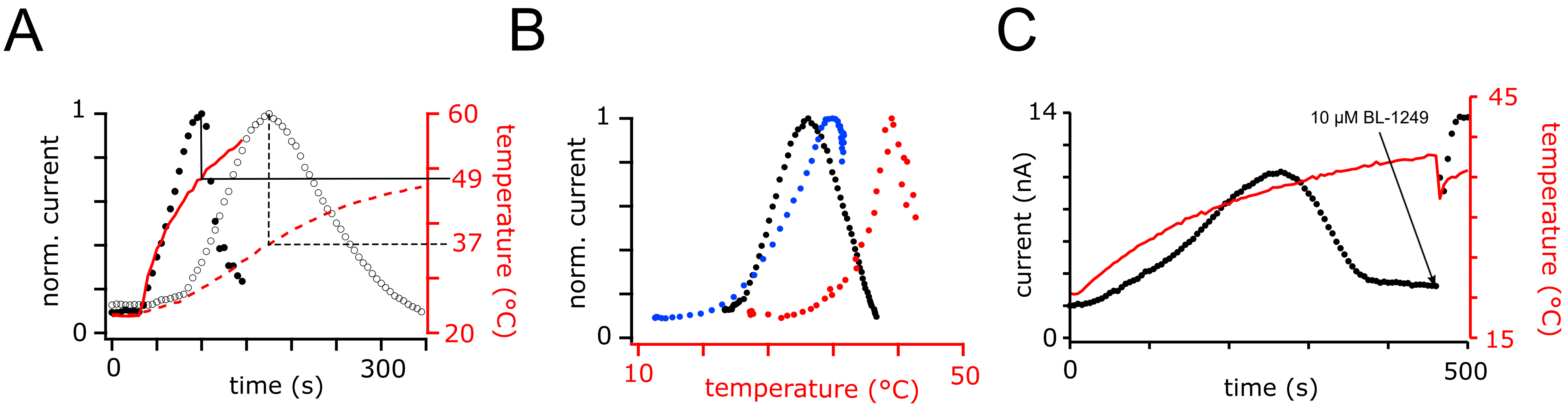

Figure S4 Temperature activation of TREK-1 channels in different recording setups. A The temperature at the current maximum depends on the temperature rise time. Faster temperature rise times result in maximal currents at higher temperature. Currents were measured with a ramp protocol and the currents at +60 mV were plotted against time. The currents were normalized to the maximal currents. Open circles/dotted lines mark currents elicited with a temperature rise time of 0.1 °C s^{-1}. Filled circles/straight lines show currents elicited with a temperature rise time of 0.25 °C s^{-1}. **B** Current changes as a function of the actual temperature for the measurements shown in A (black circles: temperature rise time 0.1 °C s^{-1}; red circles: temperature rise time 0.25 °C s^{-1}; blue circles: temperature rise time 0.1 °C s^{-1} with cooling prior to temperature elevation. **C** The TREK-1 channels are still functional even after prolongued temperature exposure as they were still activated by the application of 10 µM BL-1249.

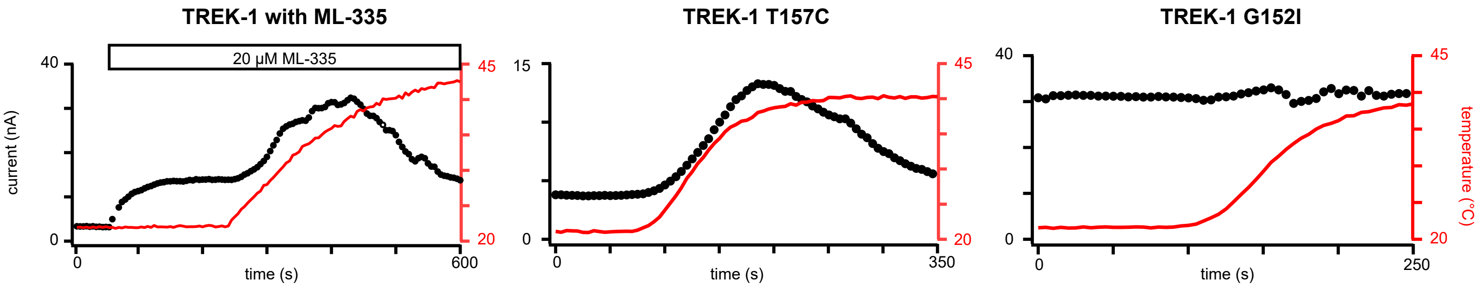

Figure S5 Activation state of the selectivity filter gate has no influence on temperature-sensitvity. Time courses of current changes upon temperature elevation in human TREK-1 channels with preactivated selectivity filter gate. Neither application of ML-335 (left panel) nor the T157C mutation (middle panel) which activates the selectivity filter gate prevented temperature induced current activation and inactivation with sustained heating. The G152I mutation (right panel) already activated the channel completely. Accordingly, no further activation of the current was possible. Currents were measured with a ramp protocol and the currents at +60 mV were plotted. Red lines: changes in bath temperature measured in °C.

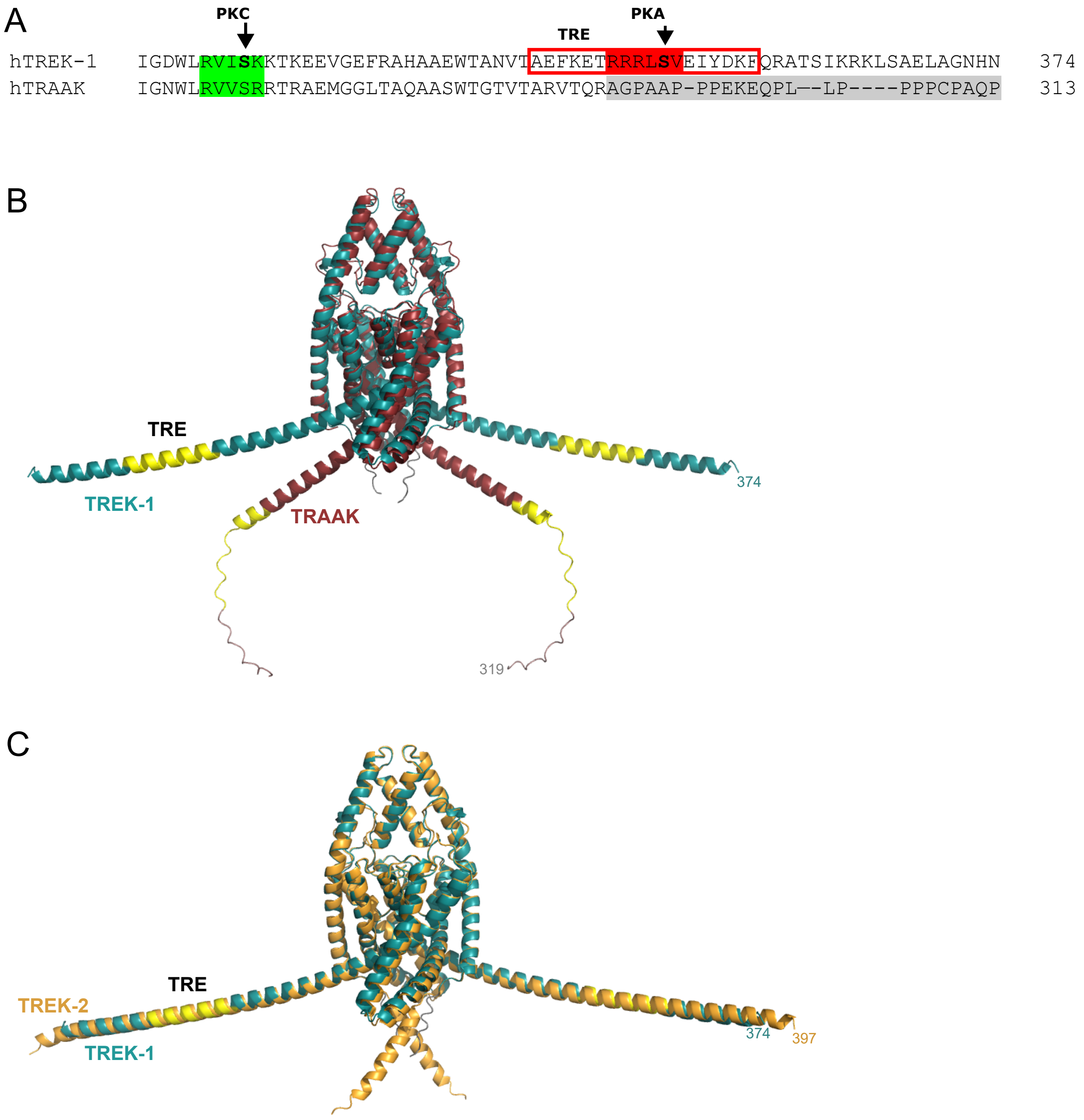

Figure S6: Only TREK C-termini contain the helical region with the PKA site and TRE. A, Sequence alignment of the proximal C-termini of hTREK-1 and TRAAK channels showing the PKC sites (green) in both channels and PKA site (red) and TRE (black box) in TREK-1 channels. The corresponding region in TRAAK channels is a proline-rich non-helical region (grey) which would dirupt the TRE. B, Alphafold 3 models of hTREK-1 (cyan) and hTRAAK (red) showing the elongated helical C-terminus with the TRE (yellow) only present on TREK-1 channels. C, Elongated helices are only present in TREK-1 and TREK-2 models, while TRAAK and representatives of other subfamilies have short helices terminating before the TRE.

Table S1 Maximal current increase by temperature elevation. Fold change of currents relative to basal current at RT were determined with the ramp protocol at +60 mV. Different control values for TREK-1 WT are given for the different data sets. P-values highlighted in bold are significant.

Channel/construct	mean	S.E.M.	p-value	n
**TREK-1 WT (channels)**	**10.5281**	**1.2371**		**68**
TREK-2	13.6708	5.249	0.532	9
TRAAK	1.8827	0.2521	**$9.2997 \cdot 10^{-9}$**	12
TALK-2	1.8808	0.2104	**$1.7857 \cdot 10^{-7}$**	9
TRESK	2.247	0.3582	**$2.006 \cdot 10^{-4}$**	5
TASK-3	1.5421	0.2082	**$2.8861 \cdot 10^{-5}$**	5
THIK-1	2.0346	0.4065	**$4.2575 \cdot 10^{-5}$**	6
TWIK-1	2.2488	0.194	**$1.8292 \cdot 10^{-8}$**	13
mTRAAK	3.4444	0.294	**0.04628**	3
TRAAK (COS7)	2.0697	0.3322	**$5.917 \cdot 10^{-4}$**	4
hTRAAK (long)	2.3696	0.259	**$2.936 \cdot 10^{-3}$**	5
TREK-1 On Cell	8.4978	1.1654	0.801	15
TREK-1 Inside-out	1.1952	0.245	**$1.2826 \cdot 10^{-4}$**	4
**TREK-1 WT (C-terminus)**	**12.4498**	**2.6471**		24
TREK1/ctTRAAK	2.53333	0.6018	**$2.462 \cdot 10^{-3}$**	4
TRAAK/ctTREK-1	10.7716	2.0139	0.6985	19
TALK-2/ctTREK-1	2.8067	0.7534	**$3.3746 \cdot 10^{-3}$**	3
TASK-3/ctTREK-1	2.13	0.4615	**$8.1741 \cdot 10^{-5}$**	6
TRAAK-TREK-1 concatamer	3.3986	0.8133	**$3.6761 \cdot 10^{-5}$**	14
TREK-1 R326Stop	2.9	0.3317	**$8.3778 \cdot 10^{-4}$**	5
TREK-1 A338Stop	1.9333	0.0667	**$7.8477 \cdot 10^{-5}$**	5
TREK-1 Q356Stop	5.6369	0.905	0.05532	10
**TREK-1 WT (Kinase)**	**6.9792**	**0.7025**		22
PKA activation (Forsk/IBMX)	3.2082	0.5433	**$6.6622 \cdot 10^{-3}$**	18
Forsk/IBMX with H89	12.8876	6.0741	0.9872	14
PKA inhibition (H89)	17.9973	6.9922	0.5531	23
PKC activation (PMA)	13.495	3.2535	0.5945	17
TREK-1 S348D (PKA phosphorylation)	2.2699	0.3425	**$7.299 \cdot 10^{-3}$**	7
TREK-1 S348A (PKA dephosphorylation)	6.0651	1.4576	0.7865	10
TREK-1 + D1R (10 µM dopamine)	2.9167	0.4307	**$1.943 \cdot 10^{-3}$**	8
**TREK-1 WT (cytoskeleton)**	**8.2884**	**1.0916**		22
Microtubule depolymerisation (Colchicine)	4.1796	0.6134	**$5.7101 \cdot 10^{-4}$**	24
Microtubule depolymerisation (Vinblastine)	3.5576	1.5832	**$8.6195 \cdot 10^{-3}$**	7
Actin depolymeristion (Cytochalasin B)	6.7393	1.3914	0.3726	7
No MAP2 binding site	4.4671	0.9157	**$2.7313 \cdot 10^{-3}$**	19
**TREK-1 WT (GTP)**	**8.015**	**1.53**		14
GTPγS	2.7588	0.6989	**$4.1464 \cdot 10^{-3}$**	6
GMP PNP	2.7404	0.6811	**0.02264**	4
**TREK-1 WT (Rb^+ Intra)**	**4.3423**	**0.8584**		3
TRAAK (Rb^+ Intra)	1.5136	0.1061	**0.01309**	6
